# Opposite serotonergic modulation of sharp waves in the dorsal and ventral hippocampus

**DOI:** 10.3389/fnsyn.2025.1701349

**Published:** 2025-11-05

**Authors:** Charalampos L. Kandilakis, Costas Papatheodoropoulos

**Affiliations:** Laboratory of Physiology, Department of Medicine, University of Patras, Rion, Greece

**Keywords:** hippocampus, dorsoventral, septotemporal, serotonin, 5-HT, sharp waves, multiunit activity, network excitability

## Abstract

Serotonin plays a crucial role in regulating hippocampal network dynamics, however, its effects on sharp wave–ripples (SPWs), a pattern fundamental for memory consolidation and emotional processing, remain incompletely understood, particularly along the dorsoventral axis. Using hippocampal slices from adult rats, we compared serotonergic modulation of SPWs and associated multiunit activity (MUA) in dorsal and ventral CA1 regions. Serotonin (1–100 μM) was applied to evaluate dose dependent and region-specific effects on SPW amplitude, duration, frequency, and neuronal firing. We found that serotonin reduces SPW amplitude in both hippocampal segments, decreases the rate of SPW occurrence in the dorsal hippocampus, and increases the rate of SPW occurrence in the ventral hippocampus, but only at relatively low concentrations. The suppressive effect on SPW amplitude is accompanied by a reduction in firing frequency during SPWs in both regions, whereas the enhancing effect of low serotonin concentrations on SPW rate in the ventral hippocampus is associated with an excitatory action on basal neuronal activity. These results reveal a region-specific, and dose-dependent serotonergic modulation of SPWs, reflecting distinct excitatory/inhibitory balances and receptor subtype distributions along the hippocampal axis. Functionally, serotonergic suppression of dorsal SPWs may regulate cognitive processes, whereas bidirectional modulation in the ventral hippocampus may fine-tune affective and stress-related responses. Our findings highlight dorsoventral specialization of serotonergic control over hippocampal network patterns, providing insights into the mechanisms of dorsoventral hippocampal specialization and the symptom heterogeneity of neuropsychiatric disorders involving serotonergic dysfunction.

## 1 Introduction

Serotonin (5-hydroxytryptamine, 5-HT) is a multifaceted neuromodulator with diverse functional roles in both physiology and pathology in the central nervous system. Physiologically, serotonin is involved in several functions including cognitive flexibility, mood and emotional regulation, stress reactivity, and modulation of sleep–wake cycle (Charnay and Léger, 2010; Pytliak et al., 2011). Furthermore, dysregulation of serotonin signaling is implicated in a range of neuropsychiatric and neurodevelopmental disorders including anxiety, depression, schizophrenia, and autism spectrum disorders (Bai et al., 2014; Müller and Homberg, 2015; Muller et al., 2016; Yamazaki et al., 2022; Lin et al., 2023). Serotonin originates from neurons in the raphe nuclei, which project widely throughout the brain (Molliver, 1987). Its actions are mediated by seven types of serotonin receptors (5-HTRs), comprising 14 receptor subtypes (Pytliak et al., 2011; McCorvy and Roth, 2015), thereby modulating both neural cell function and network activity.

A significant portion of serotonin’s influence on behavior involves the hippocampus which receives dense serotonergic innervation from the raphe nuclei (Molliver, 1987). As a critical structure for spatial navigation, episodic memory, emotional processing, affective responses, and social behavior (Bannerman et al., 2014; Blair and Fanselow, 2014; Strange et al., 2014; Okuyama et al., 2016; Eichenbaum, 2017; Shi et al., 2023), the hippocampus integrates multiple neuromodulatory inputs to support flexible cognition and behavior (Hasselmo and Giocomo, 2006; Sara, 2009). Notably, the potent effects of serotonin on hippocampal physiology are primarily mediated through its ability to regulate the excitation/inhibition (*Ε*/*Ι*) balance acting on a diversity of hippocampal neurons via multiple receptor subtypes (Bombardi et al., 2021; Kandilakis and Papatheodoropoulos, 2025). The regulation of E/I balance is a fundamental mechanism for the generation and modulation of behaviorally relevant network rhythms (Haider et al., 2006; Vogels and Abbott, 2009; Isaacson and Scanziani, 2011), and the serotonergic modulation of E/I balance dynamically shapes network activities in the hippocampus (Kocsis et al., 2006; Johnston et al., 2014; Gener et al., 2019), such as sharp waves - ripples (SPWs).

SPWs is a fundamental hippocampal network pattern that arises from synchronous activity in the CA3–CA1 circuit, depends on a finely tuned E/I balance (Giannopoulos and Papatheodoropoulos, 2013; Simeone et al., 2013; Schlingloff et al., 2014; Buzsáki, 2015; Hofer et al., 2015; Melonakos et al., 2019; Trompoukis et al., 2020), and plays a fundamental role in memory consolidation, goal-directed decision making, and off-line information processing (Buzsáki, 2015; Joo and Frank, 2018; Pfeiffer, 2020; O'Callaghan et al., 2021; Tomar et al., 2021; Kuga et al., 2023; Xie et al., 2023). Generated through coordinated network activity, SPWs enable the replay and reorganization of recent experiences, providing a substrate for long-term memory formation and the integration of information from discrete brain regions (Wilson and McNaughton, 1994; Buzsáki, 2015; Foster, 2017). Neuronal firing during SPWs is highly organized and represents spatiotemporally structured reactivations of pyramidal cells following previous experiences (Wilson and McNaughton, 1994; Buzsáki, 2015; Foster, 2017). Notably, SPWs appear altered in various psychiatric and neurodevelopmental disorders including schizophrenia (Gao et al., 2019; Nour et al., 2022; Munn et al., 2023; Ohki et al., 2024), depression (Shiozaki et al., 2023; Koketsu et al., 2024), anxiety (Çalışkan and Stork, 2019; Kuga et al., 2023), and autism spectrum disorders/fragile X syndrome (Boone et al., 2018; Pollali et al., 2021; Leontiadis et al., 2023), which are thought to result from disruption of the E/I balance in brain network (Gao and Penzes, 2015; Nelson and Valakh, 2015; Ferguson and Gao, 2018; Sohal and Rubenstein, 2019; Kirischuk, 2022).

Given that serotonin modulates both glutamatergic and GABAergic transmission, which represent fundamental components of E/I balance, often in a receptor- and region-specific manner (Kandilakis and Papatheodoropoulos, 2025), it is well-positioned to influence the generation and expression of SPWs. Evidence indicates that serotonin signaling is indeed involved in the modulation of SPWs (Ponomarenko et al., 2003; Wang et al., 2015; ul Haq et al., 2016; Jelitai et al., 2021; Cooper et al., 2025). For instance, many of the median raphe nucleus neurons are silent during SPWs and activation of these neurons inhibits SPWs (Wang et al., 2015). Also, serotonin suppresses SPWs induced by tetanic stimulation in dorsal hippocampal slices (ul Haq et al., 2016), and blockade of 5-HT3 receptors enhance hippocampal ripple oscillation (Ponomarenko et al., 2003). An inverse correlation between the serotonin’s levels and ripples has been also found recently (Cooper et al., 2025). Notably, all these studies were conducted in dorsal hippocampus preparations. Yet, a recent study shows that SSRIs selectively reduce SPWs in the ventral hippocampus (Shiozaki et al., 2023), though these drug effects could be accounted by mechanisms other than direct serotonergic modulation.

These observations have led to the idea that serotonin has an inhibitory action on SPWs (Wang et al., 2015; ul Haq et al., 2016; Jelitai et al., 2021; Shiozaki et al., 2023). However, the serotonergic modulation of SPWs may be more complicated than it seems. For instance, blockade of 5-HT1A receptors reduce the number of ripple events in the hippocampus (Ponomarenko et al., 2003), suggesting that the serotonergic modulation of SPWs may be bidirectional and receptor-specific.

Interestingly, both serotonergic modulation and SPWs display regional specialization along the dorsoventral (longitudinal) axis of the hippocampus. Both the anatomical distribution and the physiological actions of serotonergic fibers and receptors exhibit marked differences between the dorsal and ventral hippocampus, resulting in region-specific influences on neural excitability (Kandilakis and Papatheodoropoulos, 2025). More specifically, the serotonergic innervation to the ventral hippocampus is denser from the dorsal hippocampus, originates from the dorsal raphe nucleus, and exerts mainly volume transmission, while the serotonergic projection to the dorsal hippocampus originates primarily from the median raphe nucleus and exerts synapse-specific transmission (Molliver, 1987; Oleskevich and Descarries, 1990; Kandilakis and Papatheodoropoulos, 2025). Furthermore, 5-HT1 and 5-HT2 receptors display different expression in dorsal and ventral hippocampal layers (Mengod et al., 1990; Kinsey et al., 2001; Tanaka et al., 2012), and serotonin levels are higher in the ventral than in the dorsal hippocampus (Hortnagl et al., 1991). This specialization is part of a more general functional segregation that has been established along the long axis of the hippocampus, with the dorsal hippocampus more heavily involved in cognition and processing of spatial information, and the ventral hippocampus more implicated in emotional behavior and stress reactivity (Fanselow and Dong, 2010; Bannerman et al., 2014; Strange et al., 2014).

Regarding SPWs, recent studies have revealed regional specializations in the properties of this network activity along the dorsoventral axis of the hippocampus (Kouvaros and Papatheodoropoulos, 2017; De Filippo and Schmitz, 2023). SPWs generated in the dorsal hippocampus are more strongly associated with cognitive functions such as spatial navigation and episodic memory, whereas ventral SPWs may be more involved in emotional processing and stress regulation (Sosa et al., 2019; Kuga et al., 2023). For instance, dorsal and ventral SPWs preferentially activate distinct, largely non-overlapping neuronal populations in the nucleus accumbens. Notably, dorsal rather than ventral hippocampal SPWs are linked to nucleus accumbens activation during processing of spatial and reward-related information (Sosa et al., 2019). Furthermore, ventral hippocampus SPWs support stress-associated memory processing (Kuga et al., 2023). Also, in a model of Fragile X syndrome, SPWs and associated multiunit activity were impaired in the dorsal but not ventral hippocampus (Leontiadis et al., 2023).

Despite emerging evidence of serotonergic modulation of SPWs, the comparative effects of serotonin on SPWs in dorsal vs. ventral hippocampus remain poorly understood. Given this distinct anatomical and functional organization of the hippocampus along its dorsoventral axis, it is particularly important to investigate serotonergic modulation of SPWs in both the dorsal and ventral hippocampus. Understanding whether and how 5-HT differentially modulates SPWs in these regions could provide critical insights into both normal hippocampal function and the pathophysiology of neuropsychiatric disorders in which serotonergic and hippocampal function are disrupted.

In the present study we examined the effects of serotonin on dorsal and ventral hippocampal slices. We found that serotonin exerted distinct, region-specific effects. In the dorsal hippocampus, serotonin dose-dependently reduced SPWs. In contrast, in the ventral hippocampus we mainly observed an increase in SPW frequency at relatively low concentrations of serotonin, and primarily a reduction in SPW amplitude at relatively high concentrations of serotonin. These results may provide important implications for the functional organization along the hippocampal longitudinal axis. For instance, serotonergic modulation of dorsal SPWs may be related to normal cognitive processes and cognitive impairments associated with psychiatric disorders, while serotonergic modulation of SPWs in the ventral hippocampus may underlie affective and anxiety-related disturbances.

## 2 Materials and methods

### 2.1 Hippocampal slice preparation

Wistar rats 3–4 months old of both sexes were used in this study. Rats were obtained from the Laboratory of Experimental Animals of the Department of Medicine, University of Patras (licence no: EL-13-BIOexp-04). Rats treatment and all experimental procedures were performed in accordance with the European Communities Council Directive Guidelines for the care and use of Laboratory animals (2010/63/EU – European Commission), and the experimental protocol has been approved by the Protocol Evaluation Committee of the Department of Medicine of the University of Patras and the Directorate of Veterinary Services of the Achaia Prefecture of Western Greece Region (reg. Number: 187531/626, 26/06/2018). Rats were maintained under standard conditions of temperature (20–22°C) and light–dark cycle (12/12 h), and they were free access to food and water. Transverse slices were prepared from the dorsal and ventral hippocampus as previously described (Trompoukis and Papatheodoropoulos, 2020). Briefly, rats were decapitated under deep anesthesia with diethyl-ether. The brain was removed and placed in chilled artificial cerebrospinal fluid (ACSF) at a temperature of 2–4°C, equilibrated with 95% O_2_ and 5% CO_2_ gas mixture. The composition of ACSF was (in mM): 124 NaCl, 4 KCl, 2 CaCl_2,_ 2 MgSO_4,_ 26 NaHCO_3_, 1.25 NaH_2_PO_4_ and 10 glucose and a pH = 7.4. The hippocampi were removed and 550 μm thick slices were prepared from the dorsal and ventral end of the hippocampus extending between 0.5 mm and 4.0 mm from each end, using a McIlwain tissue chopper. Slices were immediately transferred to an interface type recording chamber where they were maintained for the rest of the experiment continuously perfused with ACSF at a rate of ~1.5 mL/min and humidified with 95% O_2_ and 5% CO_2_ gas mixture at a temperature of 30.0 ± 0.5°C. Slices were allowed to recover for about 1.5 h before recordings were started.

### 2.2 Electrophysiology and data analysis

**Figure 1:**
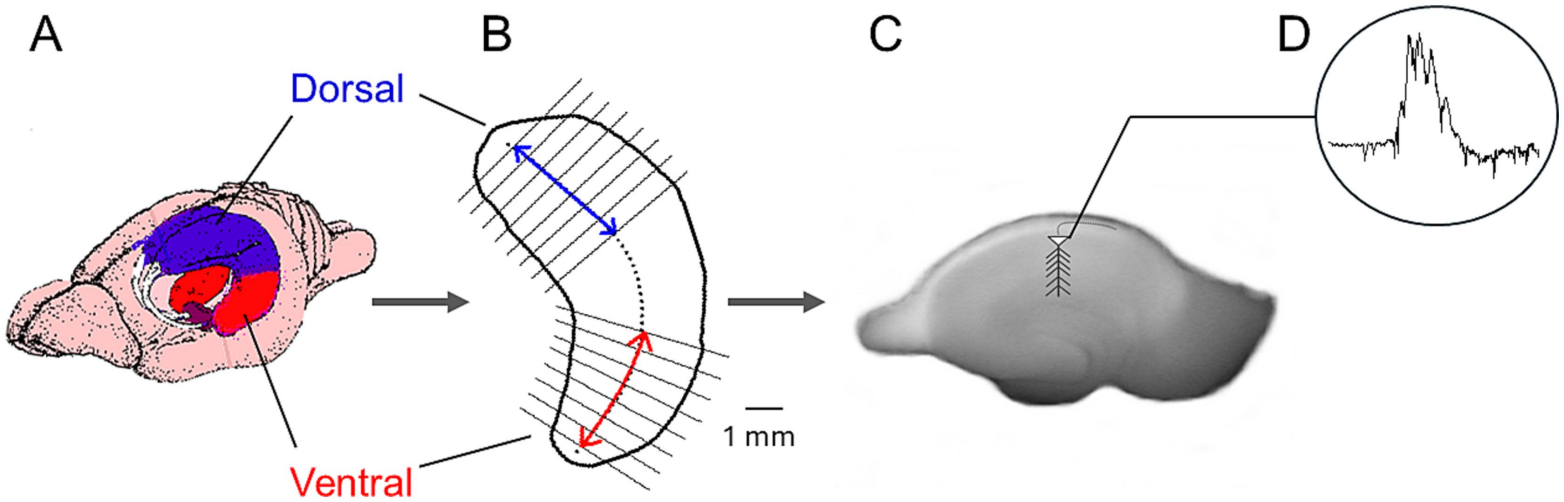
Method used to prepare dorsal and ventral hippocampal slices and record spontaneous activity. **(A)** Schematic illustration of the rat brain showing the dorsal and ventral hippocampal regions. **(B)** Outline of a hippocampus indicating the dorsal and ventral segments used to prepare transverse slices. **(C)** Photograph of a hippocampal slice with the recording electrode positioned in CA1. **(D)** Example trace of spontaneous activity.

Spontaneous field potentials were recorded from the stratum pyramidale of the CA1 hippocampal field, using carbon fiber electrodes 7 μm-thick (Kation Scientific, Minneapolis, USA). Signal was acquired and amplified X500 and then filtered at 0.5 Hz–2 kHz using Neurolog systems (Digitimer Ltd., UK), consisting of AC preamplifier (NL 104A with NL 100AK headstage), AC/DC amplifier (NL 106) and band pass filter (NL 125/6). Analog signal was digitized at 10 kHz using a CED 1401-plus interface and the Spike software (Cambridge Electronic Design, Cambridge, UK), then, stored on a computer disk for off-line analysis using the same software. We used the agonist of 5-HT (Cayman Chemical Company, USA) (1–100 μΜ). Activity consisted of sharp waves ridden by multiunit activity (MUA) (Figure 1). Events of SPWs were quantified by their amplitude measured as the voltage difference between the positive peak and the baseline. (1) The duration of single SPW events measured as the time interval between the two points of the positive phase that intersect the baseline. (2) The inter-event interval (IEI) measured as the time between successive individual SPWs. Measures of SPWs were performed after original records were down sampled (at 1 kHz) and low-pass filtered at 35 Hz. Then, individual events were detected after setting a threshold at a level where all putative events were identified as verified by visual inspection as previously described (Giannopoulos and Papatheodoropoulos, 2013). Multiunit activity (MUA) was revealed in band-pass filtered records (at 400–1.5 kHz) and was detected by setting a threshold level at a value that all putative events (i.e., negative spikes) were identified as verified by visual inspection, as previously described (Kouvaros and Papatheodoropoulos, 2017). MUA occurred between events of SPWs is called MUA-Base, and during SPWs called MUA-SPW. We quantified both MUA-Base and MUA-SPW by its frequency of occurrence (Hz). MUA-Base was measured by the frequency of MUA at steady state between consecutive events of SPWs. We measured MUA-SPW by the maximum frequency of MUA in peri-event histograms between SPWs and MUA, where we used the positive peaks of low-pass filtered SPWs as reference events (Figure 1D).

The serotonin receptor agonist 3-(2-aminpethyl)-1H-indol-5-ol, monohydrochloride (5-HT, Cayman #153–98-0, USA) was used in this study. We used the Shapiro–Wilk test to assess the normality of the value distributions for the various variables and Levene’s test to examine the equality of population variances. Drug effects in each hippocampal segment were statistically evaluated using ANOVA and either a two-tailed paired *t*-test or the corresponding non-parametric Wilcoxon signed-ranks test. The number of slices and rats (slices/rats) used in each experimental condition are provided. Group data are presented either as mean ± S. E. M. (in the text) or as box plots showing the median with the 25th and 75th quartiles, the mean, the 5th and 95th percentile, the outliers, and the normal distribution curve (in the figures).

## 3 Results

### 3.1 Comparison of SPWs and MUA in dorsal and ventral hippocampus

We compared sharp wave events (SPWs) and multi-unit activity (MUA) recorded from the dorsal and ventral hippocampus.

**Figure 2:**
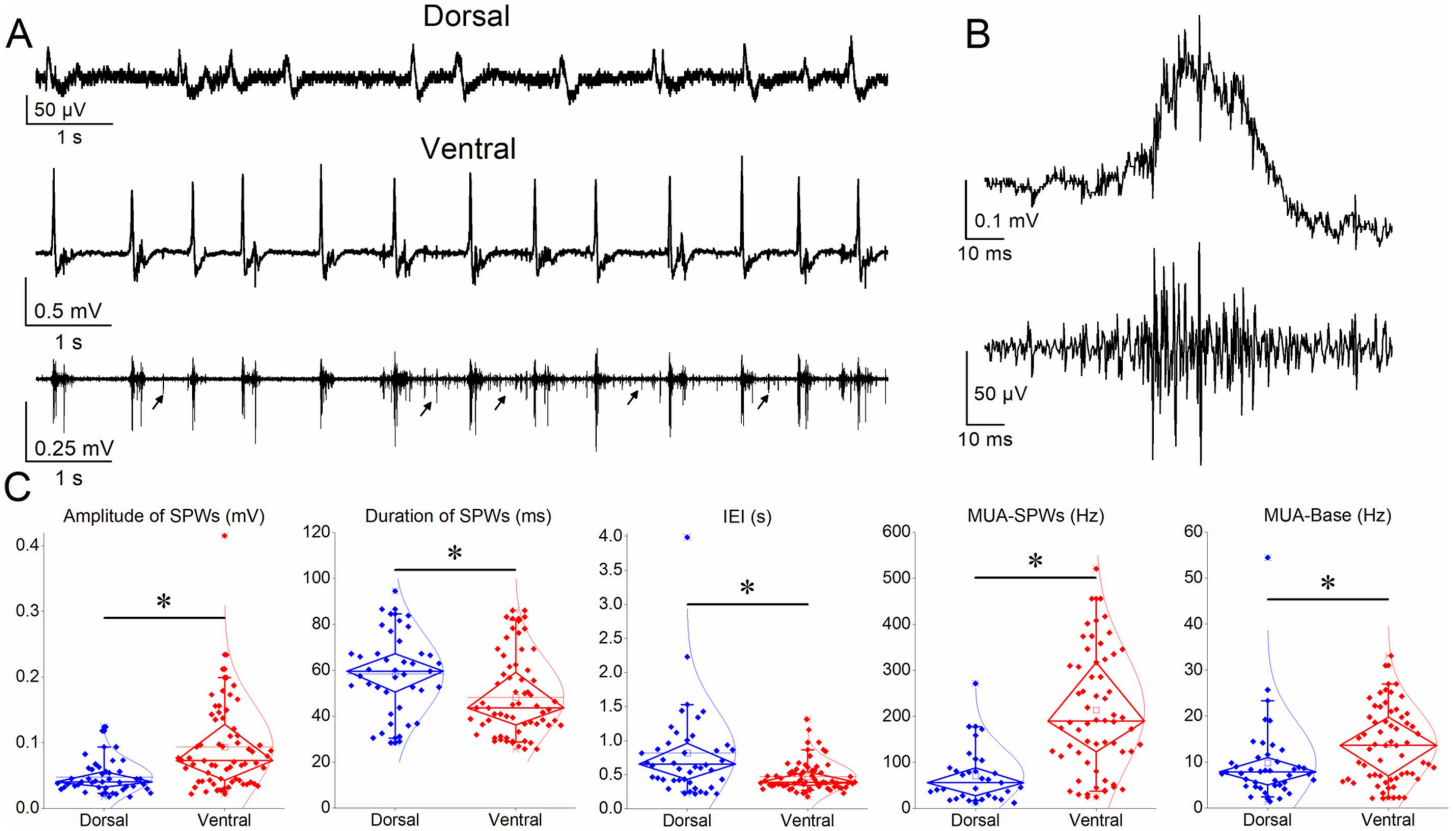
Comparison of SPWs and MUA in dorsal and ventral hippocampus. **(A)** Ten-second recordings from a dorsal (upper trace) and a ventral (middle trace) hippocampal slice illustrating SPW occurrence. The bottom trace shows the MUA associated with SPWs (MUA-SPW) in the ventral hippocampal slice, as well as the MUA occurring between SPWs (MUA-Base), occasionally indicated by arrows. **(B)** A single SPW (original trace, upper) and the corresponding MUA (MUA-SPW, lower) obtained by high-pass filtering of the original recording. **(C)** Box plots comparing the various variable between the dorsal and ventral hippocampus. Asterisks indicate statistically significant interregional differences for SPW amplitude (z = −4.56, *p* < 0.001), duration (z = −2.83, *p =* 0.005), IEI (z = −4.02, *p* < 0.001), MUA-SPW (t = −7.17, *p* < 0.001), and MUA-Base (z = −2.92, *p =* 0.003).

The ventral compared with the dorsal hippocampus generated SPWs with significantly higher amplitude (93.5 ± 8.3 μV, *n =* 64/25 vs. 47.6 ± 3.7 μV, *n =* 42/24; z = −4.56, *p* < 0.001), and shorter duration (48.3 ± 2.2 ms, *n =* 60/24 vs. 50.0 ± 0.4 ms, *n =* 37/20; z = −2.83, *p =* 0.005) (Figure 2). In addition, SPWs occurred more frequently in the ventral hippocampus (IEI: 468.8 ± 26.3 ms, *n =* 64/25) compared with the dorsal hippocampus (IEI: 820.0 ± 100.5 ms, *n =* 42/24) (z = −4.02, *p* < 0.001) (Figure 2). These findings are consistent with previous studies reporting higher-amplitude SPWs occurring at a faster rate in the ventral compared with the dorsal hippocampus (Kouvaros and Papatheodoropoulos, 2017; Trompoukis et al., 2020; Trompoukis et al., 2021; Leontiadis et al., 2023).

Regarding MUA, we found a significantly higher frequency of MUA-SPWs (MUA occurring during SPWs) in the ventral hippocampus (213.45 ± 16.84 Hz, *n =* 58/25) compared with the dorsal hippocampus (70.9 ± 10.6 Hz, *n =* 31/23) (t = −7.17, *p* < 0.001). In addition, the frequency of MUA-Base (MUA occurring between SPWs) was significantly higher in the ventral hippocampus (38.1 ± 24.2 Hz, *n =* 59/25) compared with the dorsal hippocampus (9.9 ± 1.4 Hz, *n =* 42/23; z = −2.92, *p =* 0.003).

It should also be noted that previous studies reporting greater SPW amplitude in the dorsal compared with the ventral hippocampus (Patel et al., 2013; Sosa et al., 2019) were performed in freely moving rats during sleep and awake immobility, which makes a significant difference compared with our isolated slice preparation. Transverse slices lack both extrahippocampal inputs and the intrinsic longitudinal connections that extend along the septotemporal axis of the hippocampus (Swanson et al., 1978; Ishizuka et al., 1990), which are preserved *in vivo* and are thought to contribute critically to the modulation of SPWs across hippocampal segments (Sullivan et al., 2011; Patel et al., 2013). Thus, SPWs recorded in slices most likely reflect the local dynamics of dorsal or ventral hippocampal circuits in isolation, whereas in vivo recordings capture the integrated activity of longitudinally connected networks. These methodological differences may underlie the apparent discrepancy in dorsoventral SPWs amplitude observed between in vivo and *in vitro* conditions.

### 3.2 Distinct effects of serotonin between the dorsal and ventral hippocampus

**Figure 3:**
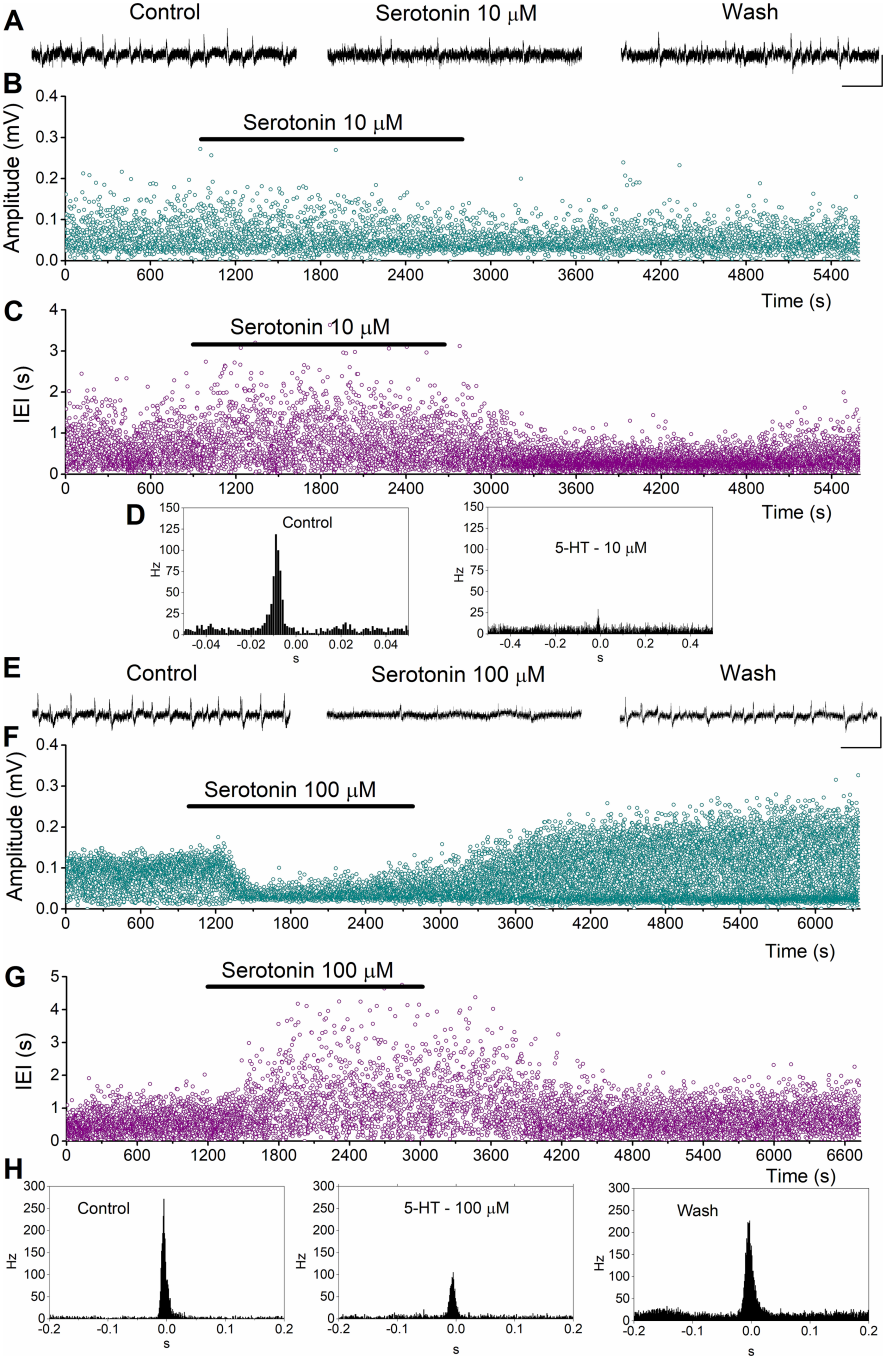
Examples of the effects of serotonin on SPWs and MUA in the dorsal hippocampus. **(A–D)** Effects of 10 μM serotonin. Continuous recordings **(A)**, instantaneous histograms of SPW amplitude **(B)**, inter-event interval (IEI, **C)**, and peri-event histograms of MUA triggered by SPW peak positivity **(D)** are shown. **(E–H)**. Effects of 100 μM serotonin. Continuous recordings **(E)**, instantaneous histograms of SPW amplitude **(F)**, inter-event interval (IEI, **G**), and peri-event histograms of MUA triggered by SPW peak positivity **(H)** are shown. Calibration bars in continuous recordings: 0.1 mV, 2 s.

**Figure 4:**
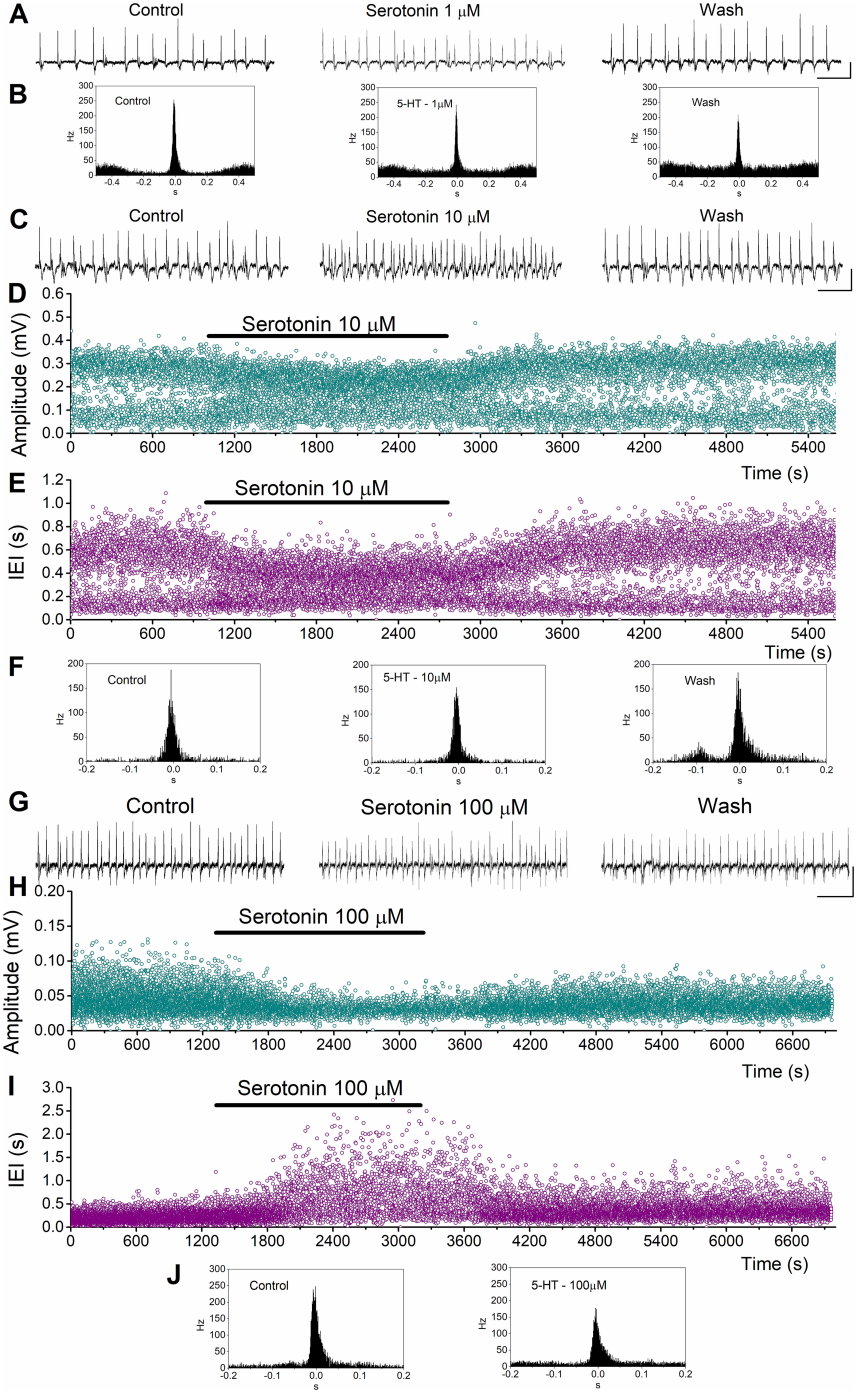
Examples of the effects of serotonin on SPWs and MUA in the ventral hippocampus. **(A,B)** Effects of 1 μM serotonin. Continuous recordings **(A)**, and peri-event histograms of MUA triggered by SPW peak positivity **(B)** are shown. **(C–F)** Effects of 10 μM serotonin. Continuous recordings **(C)**, instantaneous histograms of SPW amplitude **(D)**, inter-event interval (IEI, **E**), and peri-event histograms of MUA triggered by SPW peak positivity **(F)**. **(G–J)** Effects of 100 μM serotonin. Continuous recordings **(G)**, instantaneous histograms of SPW amplitude **(H)**, inter-event interval (IEI, **I**), and peri-event histograms of MUA triggered by SPW peak positivity **(J)**. Calibration bars in continuous recordings: 0.1 mV, 2 s.

**Figure 5:**
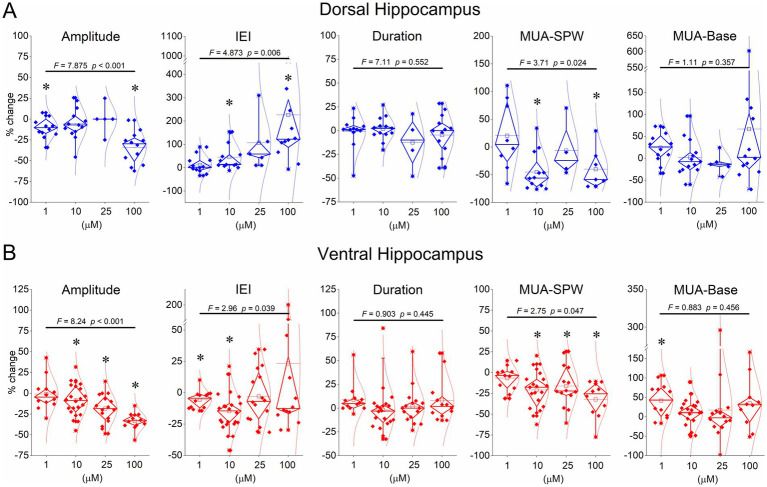
Summary data showing the effects of serotonin on SPW amplitude, IEI, MUA-SPW, and MUA-Base. Breaks in the Y-axis in some graphs are shown for clarity. The results of the ANOVA are indicated at the top of the plots. Asterisks denote statistically significant drug effects (*p* < 0.05). The statistical evaluation of serotonin’s effects at concentrations of 1 μM, 10 μM, 25 μM, and 100 μM is as follows: Dorsal hippocampus - Amplitude: z = −2.197, *p =* 0.028; t = −1.492, *p =* 0.161; z = −0.944, *p =* 0.345; t = −4.029, *p =* 0.002 - IEI: z = 0.711, *p =* 0.477; z = 3.040, *p =* 0.002; z = 2.023, *p =* 0.043; t = 2.367, *p =* 0.037 – Duration: z = −0.580, *p =* 0.532; t = 0.184, *p =* 0.857; z = −0.730, *p =* 0.465; z = −0.786, *p =* 0.432 – MUA-SPW: z = −0.280, *p =* 0.779; z = −2.903, *p =* 0.004; z = −0.730, *p =* 0.465; t = −2.192, *p =* 0.071 - MUA-Base: t = 1.219, *p =* 0.248; z = −0.804, *p =* 0.422; z = −1.753, *p =* 0.080; z = −0.392, *p =* 0.695. Ventral hippocampus: Amplitude: t = 0.515, *p =* 0.617; t = −2.484, *p =* 0.022; z = −2.999, *p =* 0.003; z = −3.059, *p =* 0.002 - IEI: t = −2.920, *p =* 0.014; z = −3.393, p < 0.001; z = −0.672, *p =* 0.501; z = −0.392, *p =* 0.695; Duration: t = 1.871, *p =* 0.088; t = −1.142, *p =* 0.268, t = 0.232, *p =* 0.819; t = 1.075, *p =* 0.305 – MUA-SPW: t = −0.751, *p =* 0.470; t = −4.314, p < 0.001; z = −2.103, *p =* 0.035; t = −3.593, *p =* 0.006 - MUA-Base: t = 2.835, *p =* 0.016; t = 0.658, *p =* 0.518; z = −0.596, *p =* 0.551; t = 1.290, *p =* 0.229. Typically, the effects of serotonin were reversible upon washout of the drug in the dorsal hippocampus: Amplitude: z = −1.376, *p =* 0.169; t = 0.076, *p =* 0.941; z = −0.674, *p =* 0.500; t = 1.575, *p =* 0.146 - IEI: z = −1.274, *p =* 0.203; z = −0.943, *p =* 0.345; z = −0.674, *p =* 0.500; t = −1.111, *p =* 0.293; Duration: t = 1.526, *p =* 0.161; t = 0.313, *p =* 0.760; z = −0.730, *p =* 0.465; t = 0.590, *p =* 0.568 – MUA-SPW: t = 0.147, *p =* 0.889; z = −1.255, *p =* 0.209; z = 0.000, *p =* 1.000; t = −1.895, *p =* 0.107 - MUA-Base: t = 1.833, *p =* 0.100; z = −0.804, *p =* 0.422; z = −1.461, *p =* 0.144; z = −1.334, *p =* 0.182. Ventral hippocampus: Amplitude: z = −0.178, *p =* 0.859; z = −0.035, *p =* 0.972; z = −0.672, *p =* 0.501; z = −0.622, *p =* 0.534 – IEI: t = 1.219, *p =* 0.251; z = −0.081, *p =* 0.935; z = −2.017, *p =* 0.044; t = 0.794, *p =* 0.446 - Duration: z = −1.824, *p =* 0.068; t = −0.459, *p =* 0.652; t = 1.253, *p =* 0.229; t = 2.643, *p =* 0.025 - MUA-SPW: t = −1.977, *p =* 0.076; t = −0.644, *p =* 0.528; z = −1.013, *p =* 0.311; t = −1.368, *p =* 0.209 - MUA-Base: t = 0.673, *p =* 0.516; z = −0.926, *p =* 0.355; z = −1.570, *p =* 0.116; t = 1.214, *p =* 0.259.

We perfused dorsal and ventral hippocampal slices with various concentrations of serotonin (1 μM, 10 μM, 25 μM, and 100 μM) and observed its effects on SPWs and MUA. Examples of these actions are shown in Figure 3 for the dorsal hippocampus and in Figure 4 for the ventral hippocampus. In the dorsal hippocampus, serotonin produced a significant reduction in SPW amplitude (average change −14.38 ± 3.24%, z = −4.158, *p* < 0.001, *n =* 42/28) and rate of occurrence (average change of IEI 94.76 ± 26.0%, z = −4.49, *p* < 0.001, *n =* 42/28), without significantly affecting the duration of individual SPW events (average change −2.31 ± 2.91%, t = 1.266, *p =* 0.213, *n =* 41/28) (Figure 5). Furthermore, these effects were accompanied by a significant reduction in MUA-SPW frequency (average change −22.1 ± 9.42%, z = −3.175, *p =* 0.002, *n =* 29/28), but not in MUA-Base (average change 25.1 ± 15.76%, z = −0.388, *p =* 0.698, *n =* 30/28), suggesting that serotonin disrupts neuronal firing during SPWs without significantly affecting background neuronal excitability. The statistical results of serotonin’s effects in the dorsal hippocampus at each concentration are presented separately in Figure 5.

Application of serotonin to ventral hippocampal slices revealed a response pattern that partially differed from that observed in dorsal slices (Figures 4, 5). Specifically, as in the dorsal hippocampus, serotonin significantly reduced the amplitude of SPWs (average change −14.43 ± 2.57%, z = −4.456, *p* < 0.001, *n =* 62/23) and MUA-SPW (average change −18.97 ± 3.03%, z = −4.81, *p* < 0.001, *n =* 53/21), without significantly affecting SPW duration (average change 3.31 ± 2.75%, z = −0.02, *p =* 0.986, *n =* 60/23). Furthermore, serotonin significantly affected the rate of SPW occurrence (z = −3.25, *p =* 0.001, *n =* 62/23), an effect that was mainly due to an enhancing effect produced at relatively low serotonin concentrations (1–10 μM, average change −11.50 ± 2.34%, z = −4.257, *p* < 0.001, *n =* 34/18). This enhancing effect was accompanied by a significant increase in the baseline excitation level (by 21.0 ± 7.3%, z = −2.39, *p =* 0.017, *n =* 12/7; see Figure 5B). The statistical results of serotonin’s effects in the ventral hippocampus at each concentration are presented separately in Figure 5.

These findings suggest that serotonin consistently reduces activity in the dorsal hippocampus, whereas it exerts a biphasic effect in the ventral hippocampus. Relatively low serotonin levels accelerate SPW occurrence and enhance baseline excitation in the ventral hippocampus, whereas higher concentrations suppress activity.

## 4 Discussion

In this study, we examined the effects of serotonin on SPWs and MUA in dorsal and ventral hippocampal slices. Based on the results obtained, the following pattern of serotonin action across the two hippocampal segments emerges: serotonin reduces SPW amplitude in both segments, decreases the rate of occurrence only in the dorsal hippocampus, and increases the rate of occurrence in the ventral hippocampus, but only at relatively low concentrations. The suppressive effect on SPW amplitude is accompanied by a reduction in firing frequency during SPWs (MUA-SPW) in both regions, a mechanism that may contribute to the observed amplitude decrease, whereas the enhancing effect of low serotonin concentrations on SPW occurrence was associated with an excitatory action on basal neuronal activity (MUA-Base). These findings reveal a clear region-specific and dose-dependent modulation of SPW dynamics, suggesting differential roles of serotonergic modulation in dorsal and ventral hippocampal networks. To our knowledge, this is the first direct comparison of serotonergic modulation of SPWs in isolated dorsal and ventral hippocampus.

Most prior studies on serotonergic regulation of SPWs have focused on the dorsal hippocampus. The inhibitory effects we observed dorsally are consistent with earlier *in vitro* and *in vivo* reports showing serotonin-dependent suppression of SPW amplitude and rate (ul Haq et al., 2016; Jelitai et al., 2021; Cooper et al., 2025). More specifically, the reduction in the amplitude and rhythm of SPWs by dorsal hippocampal serotonin that we found here is generally in agreement with previous in vitro (ul Haq et al., 2016) and in vivo studies (Cooper et al., 2025). The complete suppression previously reported at 20 μM serotonin in dorsal slices (ul Haq et al., 2016) may reflect methodological differences, as those SPWs were evoked by tetanic stimulation, whereas our study examined spontaneously generated events.

The enhancing effect of low serotonin in the ventral hippocampus contrasts with *in vivo* data showing that selective serotonin reuptake inhibitors (SSRIs) suppress ventral SPWs (Shiozaki et al., 2023). Methodological differences likely account for this discrepancy. In the intact hippocampus, dorsal and ventral segments are connected via intrinsic longitudinal pathways (Swanson et al., 1978; Yang et al., 2014), and SPWs are typically generated dorsally and propagate ventrally (Sullivan et al., 2011; Patel et al., 2012). Thus, serotonergic suppression in the dorsal hippocampus *in vivo* would be expected to secondarily reduce ventral SPWs via polysynaptic longitudinal or extrahippocampal circuits. In addition, SSRIs exhibit affinity also for dopamine and noradrenaline transporters (Tatsumi et al., 1997) that may further influence their effects on SPW activity.

Our data, demonstrating facilitation in the ventral region at low 5-HT, and suppression in both regions at high 5-HT, are also compatible with those of a recent study that reveals an inverted-U curve of serotonin which dynamically modulates SPW timing and power, with intermediate 5-HT levels favoring ripple generation during ultraslow (~0.01 Hz) endogenous serotonin oscillations (Cooper et al., 2025). Therefore, the present findings provide further information about possible distinct regional-related modulation of SPWs, pointing toward a receptor subtype- and dose-dependent profile of serotonergic modulation, as also suggested by regional differences in interneuron and pyramidal cell sensitivity (Kandilakis and Papatheodoropoulos, 2025). Accordingly, the contrasting effects of serotonin on SPWs across the dorsal and ventral hippocampus likely reflect differences in 5-HT receptor subtype expression between the two hippocampal regions (Tanaka et al., 2012).

More specifically, inhibitory receptors such as 5-HT1A and 5-HT1B, which couple to G_i/o_ proteins and activate GIRK potassium channels causing hyperpolarization and reduced neuronal excitability (Beck and Goldfarb, 1985; Schmitz et al., 1995; Kasamo et al., 2001); see also review by (Kandilakis and Papatheodoropoulos, 2025), are expressed in both the dorsal and ventral hippocampus (Berumen et al., 2012; Tanaka et al., 2012) and may play a key role in these suppressive effects. 5-HT1A receptors inhibit both pyramidal neurons and interneurons in the two regions; however, in the ventral hippocampus, 5-HT1A receptor activation can become functionally excitatory due to disinhibition via GABAergic modulation (Mlinar and Corradetti, 2018; Kandilakis and Papatheodoropoulos, 2025).

Conversely, the enhancement of activity observed at low serotonin concentrations in the ventral hippocampus may result from the activation of excitatory receptors such as 5-HT2A/2C, 5-HT4, and 5-HT7 (Alves et al., 2004; Tanaka et al., 2012; Zareifopoulos and Papatheodoropoulos, 2016). The 5-HT2A/2C receptors facilitate the release of both glutamate and GABA through G_q/11_-mediated increases in Ca^2+^ conductance and decreases in K^+^ conductance (reviewed in Kandilakis and Papatheodoropoulos, 2025). Both glutamate and GABA are fundamental components in the generation of SPWs (Buzsáki, 2015). The 5-HT4 (Andrade and Chaput, 1991; Torres et al., 1995; Chapin et al., 2002; Mlinar et al., 2006) and 5-HT7 receptors (Gill et al., 2002; Tokarski et al., 2003; Andreetta et al., 2016) increase intracellular cAMP levels, leading to excitation of pyramidal neurons and facilitation of neuronal firing. Therefore, the enhancing effects may be attributed to differences in receptor expression and/or the strength of downstream signaling pathways rather than to receptor affinity per se. Interestingly, 5-HT2A/2C, 5-HT4, and 5-HT7 receptors are highly expressed in the CA3 region of the ventral hippocampus (Andrade and Chaput, 1991; Roychowdhury et al., 1994; Gill et al., 2002; Tokarski et al., 2003; Ohmura et al., 2015). Given that SPWs are predominantly initiated in CA3 and propagate to CA1 (Buzsáki, 2015), activation of these receptors in CA3 could contribute to the acceleration of activity observed in the ventral hippocampus.

SPWs are, by definition, synchronous population events generated by the coordinated activity of neurons in the CA3–CA1 circuit (Buzsáki, 2015). The degree of synchrony and the amplitude of each SPW vary from event to event, likely reflecting both the number of participating neurons and their temporal coordination. Thus, while the field SPW potential reflects the summed synaptic currents, the MUA-SPW directly represents the intensity and temporal coincidence of neuronal discharges contributing to that population event. Evidence supporting the relationship between MUA and synchrony includes the observation that MUA frequency peaks within a few milliseconds of the SPW maximum (Buzsáki, 2015; Kouvaros and Papatheodoropoulos, 2017); present results), and that the number of action potentials during SPWs determines ripple amplitude and population synchrony (Schlingloff et al., 2014). Therefore, MUA-SPW provides a quantitative measure of how strongly neurons fire together during each SPW. A reduction in MUA-SPW, as observed with serotonin, thus indicates weaker synchrony and/or reduced neuronal participation in each SPW. Therefore, quantifying MUA-SPW complements field-potential analysis by providing a more direct measure of neuronal coactivation during SPWs.

Finally, baseline network excitability differences may also contribute to the observed serotonin effects. The ventral hippocampus is intrinsically more excitable, exhibiting a lower inhibition compared to the dorsal region (Papatheodoropoulos, 2018), thereby exhibiting a different set point of E/I balance compared to the dorsal hippocampus. This differential “background” of E/I balance likely shapes the distinct responses to serotonin. Low concentrations of 5-HT may preferentially recruit excitatory networks ventrally, possibly via 5-HT2A/2C or 5-HT4 receptor activation (Mlinar et al., 2006; Li et al., 2018), thereby facilitating SPW generation. Activation of ventral 5-HT3 receptors may enhance SPWs by modulating interneuron activity that orchestrates hippocampal network rhythms. However, at higher concentrations, serotonergic actions may shift the E/I balance toward inhibition, reducing SPWs. In the dorsal hippocampus, where the baseline inhibitory tone is higher and the network more tightly regulated, serotonin consistently suppresses network activity, potentially by enhancing GABAergic tone and suppressing pyramidal cell output.

These results have significant implications for understanding the functional role of serotonin in hippocampal physiology and its potential contribution to neuropathology. Given the role of dorsal hippocampus in spatial memory and cognitive functions, serotonergic suppression of SPWs in this region could modulate memory consolidation, navigation, and decision-making (Joo and Frank, 2018). For instance, strong suppression of SPWs by 5-HT is consistent with a gating role for 5-HT in cognitive processing and limiting memory consolidation when serotonergic tone is high. In contrast, the ventral hippocampus, more involved in emotion and stress, may rely on serotonin to modulate affective responses and stress-related memory encoding (Ishikawa and Nakamura, 2006; Kuga et al., 2023). The dose-dependent bidirectional modulation in the ventral region may serve to fine-tune emotional processing, increasing encoding at low levels of serotonin, for instance during mild arousal, and suppressing overactivation at higher levels, for instance during heightened stress.

The different effects of serotonin in dorsal and ventral regions could also contribute to the regional susceptibility observed in psychiatric conditions. Dysregulation of both serotonin signaling (Bai et al., 2014; Müller and Homberg, 2015; Muller et al., 2016; Yamazaki et al., 2022; Lin et al., 2023; Yu et al., 2025) and hippocampal SPWs (Gao et al., 2019; Jones et al., 2019; Munn et al., 2023; Ohki et al., 2024) has been implicated in schizophrenia, depression, autism spectrum disorders, and Alzheimer’s disease. Disorders with dorsal hippocampal dysfunction, such as memory impairment in Alzheimer’s disease or schizophrenia, might, in part, reflect altered serotonergic gating of SPWs and associated network replay. For example, in schizophrenia, cognitive deficits are linked to dorsal hippocampal serotonin dysregulation, while positive symptoms (hallucinations, delusions), negative symptoms (apathy, avolition, asociality), and affective comorbidities (anxiety, mood instability) involve dysregulation of the ventral hippocampus serotonergic system (Kandilakis and Papatheodoropoulos, 2025). Abnormal serotonergic modulation of SPWs in these regions may contribute to symptom heterogeneity, such as impaired memory formation (via dorsal suppression) or maladaptive emotional encoding (via altered ventral SPWs). For instance, disruption of serotonergic modulation in the dorsal hippocampus could disrupt the accuracy of SPWs, leading to poor memory encoding and cognitive dysfunction, contributing to cognitive impairment in Alzheimer’s disease or schizophrenia (Adams et al., 2008; Chen et al., 2024). On the other hand, dysregulated serotonin levels may lead to hyperexcitability of the ventral hippocampus network, excessive SPWs, and aberrant emotional significance processing, possibly contributing to positive symptoms such as hallucinations and delusions in schizophrenia, and emotional dysregulation in depression (McHugo et al., 2019; Hernandes et al., 2021). Accordingly, symptom- and region-specific interventions targeting 5-HT signaling may maximize therapeutic efficacy and minimize side effects.

In summary, these results reveal that serotonin exerts distinct modulation of hippocampal SPW events in the dorsal and ventral hippocampus. These findings highlight the complex, region-specific serotonergic modulation of hippocampal network patterns and suggest a mechanistic basis for the differential roles of serotonin in cognitive and affective functions linked to the dorsal and ventral hippocampus, respectively. Further, these serotonergic actions in the intrinsic hippocampal pattern point to the possible distinct dorsoventral roles of serotonin dysregulation in neuropsychiatric disorders. Further work clarifying receptor-specific contributions and network interactions will help to better understand how serotonergic modulation of SPWs shapes memory and emotion in health and disease.

## Data Availability

The raw data supporting the conclusions of this article will be made available by the authors, without undue reservation.
